# Supplementary material for: Sub-nanometer depth resolution and single dopant visualization achieved by tilt-coupled multislice electron ptychography
Source: Nat Commun. 2025 Jan 31;16:1219. doi: 10.1038/s41467-025-56499-1 (PMC11785980; doi:10.1038/s41467-025-56499-1)
Supplement: Supplementary file 1 — Supplementary Information [file 41467_2025_56499_MOESM1_ESM.pdf]

Supplementary Information for

Sub-nanometer depth resolution and single dopant visualization achieved by tilt-coupled multislice electron ptychography

Zehao Dong, Yang Zhang, Chun-Chien Chiu, Sicheng Lu, Jianbing Zhang, Yu-Chen Liu, Suya Liu, Jan-Chi Yang, Pu Yu, Yayu Wang, Zhen Chen*

Corresponding author: zhen.chen@iphy.ac.cn

The PDF file includes:

Supplementary Figures 1 to 14
Supplementary Table 1

Other Supplementary Materials for this manuscript include the following:

Supplementary Movie 1

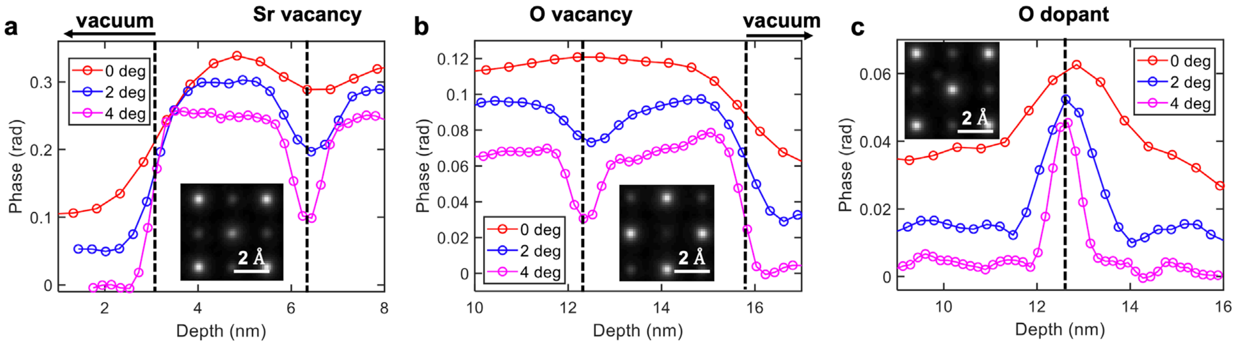

Supplementary Fig. 1| Simulation of imaging various atomic defects. a, Phase-depth curves of the Sr column with a vacancy, reconstructed using maximum tilt angles of 0°, 2°, 4°. The upper surface of the sample and the Sr vacancy position are marked with dashed lines. Inset shows phase image of the slice at the depth of the Sr vacancy. **b-c**, Similar phase-depth curves for O vacancies and O dopants, under the same tilt conditions.

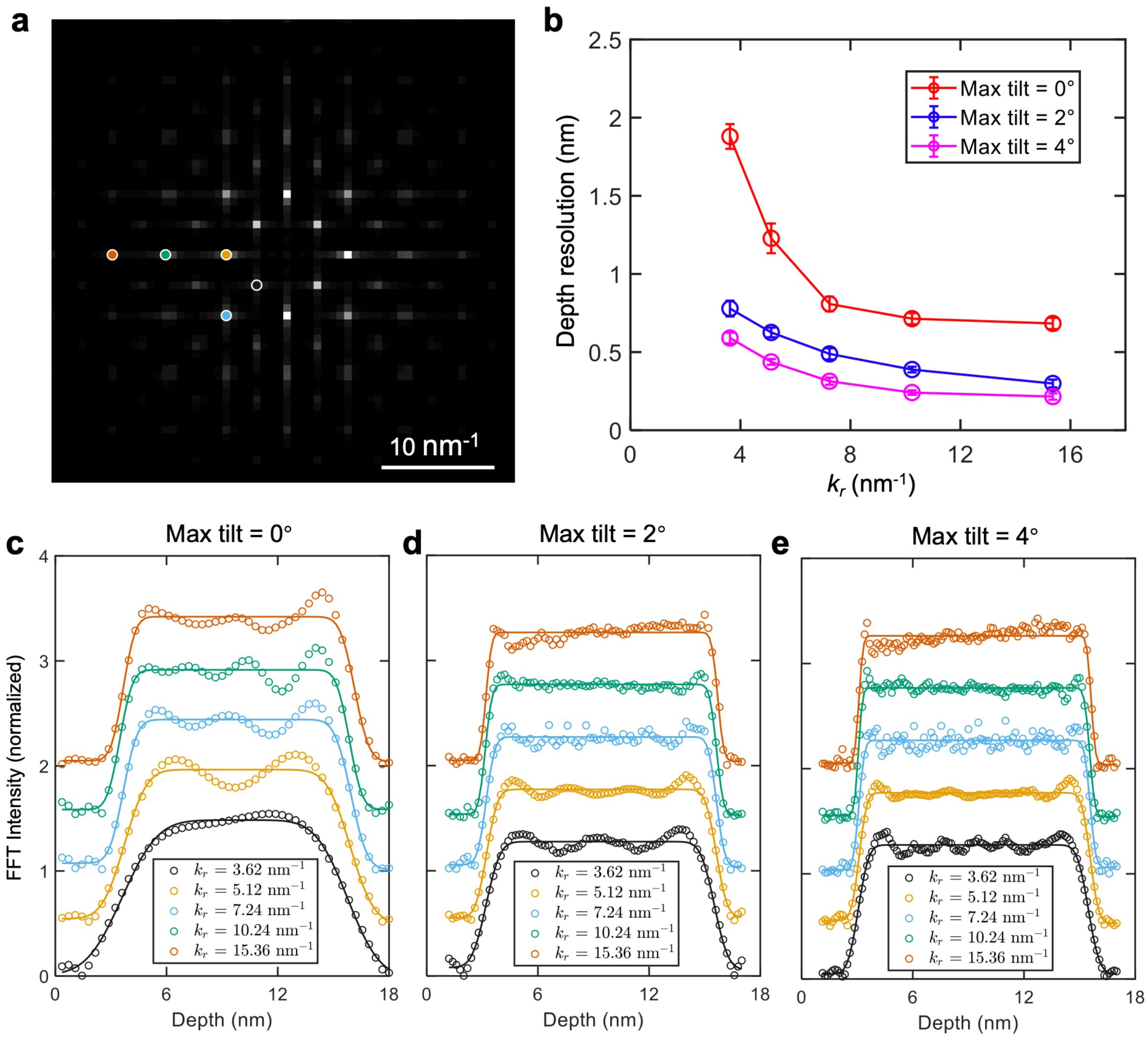

Supplementary Fig. 2| Depth resolution of TCMEP versus spatial frequency in simulations. **a**, Typical FFT image of a reconstructed $SrTiO_3$ crystal in simulations. **b**, Depth resolution for each Bragg peak under different tilt conditions. **c-e**, Depth profiles for five lateral spatial frequencies indicated by circles in panel **a**, corresponding to maximum tilts of 0° (**c**), 2° (**d**), and 4° (**e**), respectively. Gaussian error functions are used to fit the data points and determine depth resolutions at each lateral frequency. For each reconstruction, the step edges become sharper for higher-order Bragg peaks, indicating better depth resolution at higher spatial frequencies. As the tilt angle increases, the step edges for all Bragg peaks sharpen universally, validating the improved depth resolution in TCMEP and the expanded boundary for 3D information transfer. Error bars are derived from residuals of curve fitting.

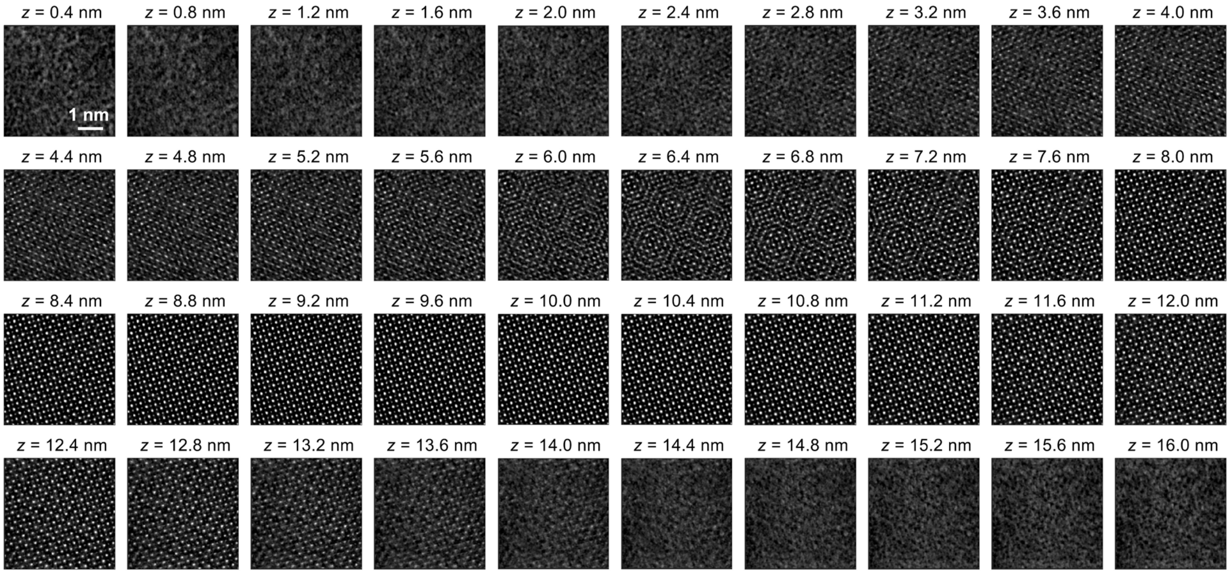

Supplementary Fig. 3| Full reconstructed dataset for Fig. 3a in the main text. Maximum tilt angle is 0°, with corresponding depth annotated above each slice. Scale bar remains identical for each image.

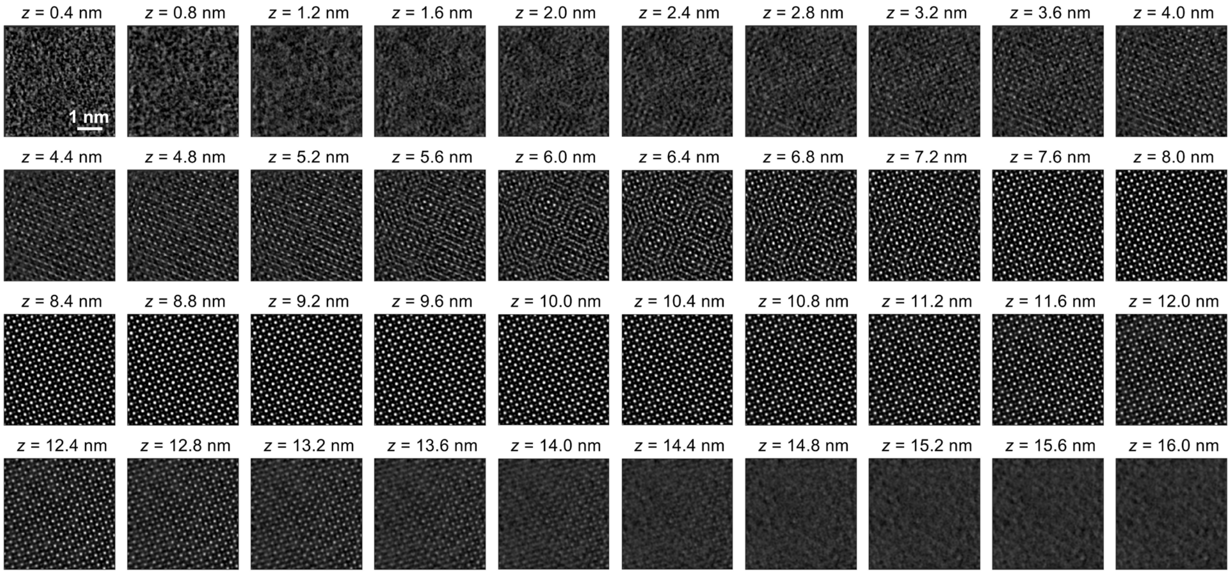

Supplementary Fig. 4| Full reconstructed dataset for Fig. 3b in the main text. Maximum tilt angle is 2°, with corresponding depth annotated above each slice. Scale bar remains identical for each image.

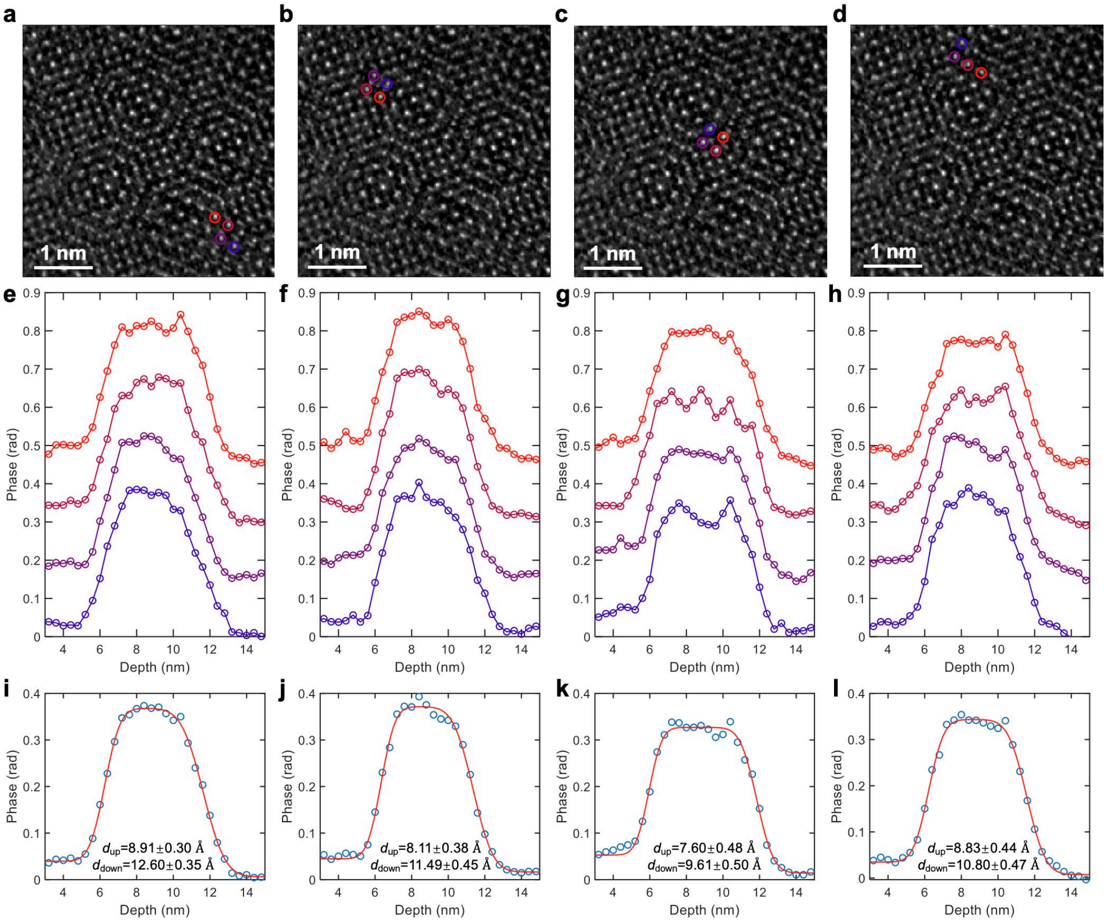

Supplementary Fig. 5| Atomic columns for determining the depth resolution with a maximum tilt of 2°. a-d, Moiré patterns formed by the twisted bilayer $SrTiO_3$ sample. Circles highlight atomic columns from the bottom layer located near the edge of the Moiré pattern, which are well separated in the lateral dimension from other atoms in the top layer. **e-h**, Phase-depth curves of the selected atoms in panels **a-d** respectively, shifted vertically for clarity. **i-l**, Averaged phase-depth curves fitted with $\boldsymbol{y = A\,\mathrm{erf}\left[(x-\mu)/\sqrt{2}\sigma\right] + B}$ on both the top and bottom surfaces, where $\boldsymbol{\mathrm{erf}(z) = \int_0^z \exp(-t^2)\,\mathrm{d}t}$ is the Gaussian error function. Notably, the depth resolution from the top surface (i.e., from the interface) is slightly better than that from the bottom surface, which is referenced in the main text. Error bars are derived from residuals of curve fitting.

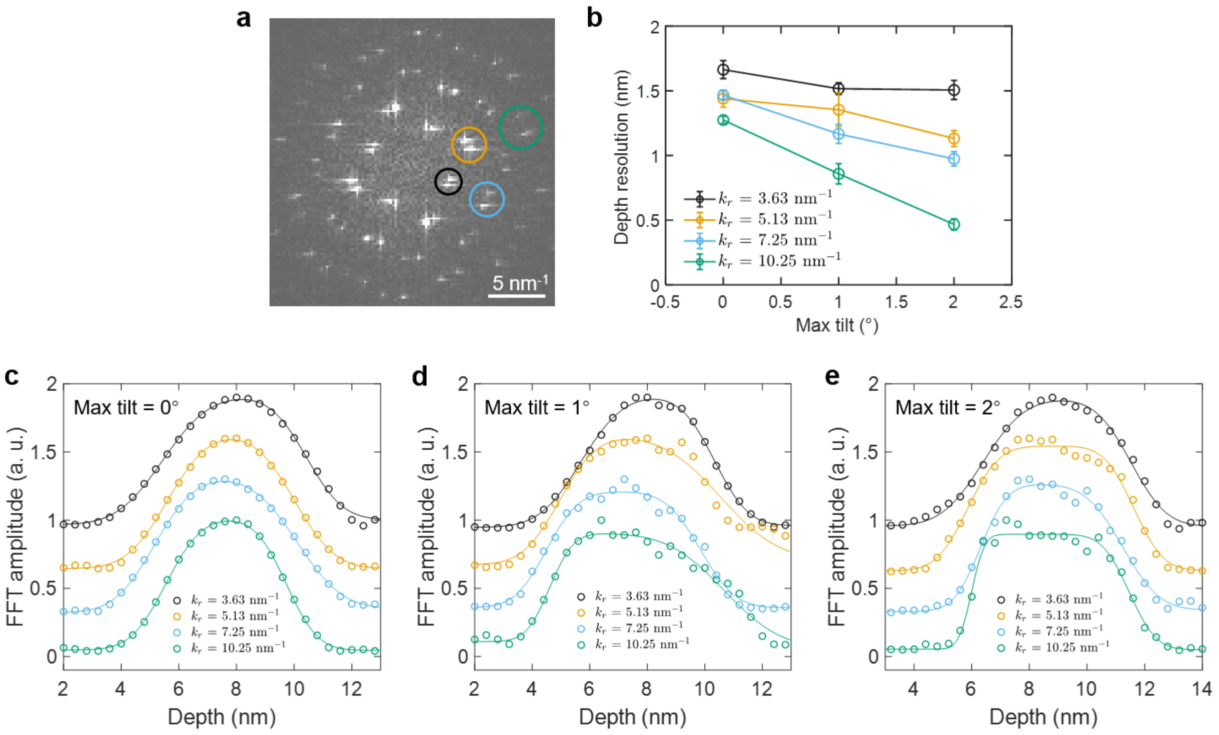

Supplementary Fig. 6| Depth resolution versus lateral spatial frequency for twisted $SrTiO_3$. **a**, Fourier transformation of a slice containing the Moiré pattern, exhibiting distinct Bragg peaks. **b**, Depth resolution for each Bragg peak under different tilt conditions, extracted following the procedure in panels **c-e**. **c-e**, Intensity of Bragg peaks versus depth, fitted with Gaussian error functions to extract depth resolution for maximum tilt angles of 0° (**c**), 1° (**d**), and 2° (**e**). Error bars are derived from residuals of curve fitting.

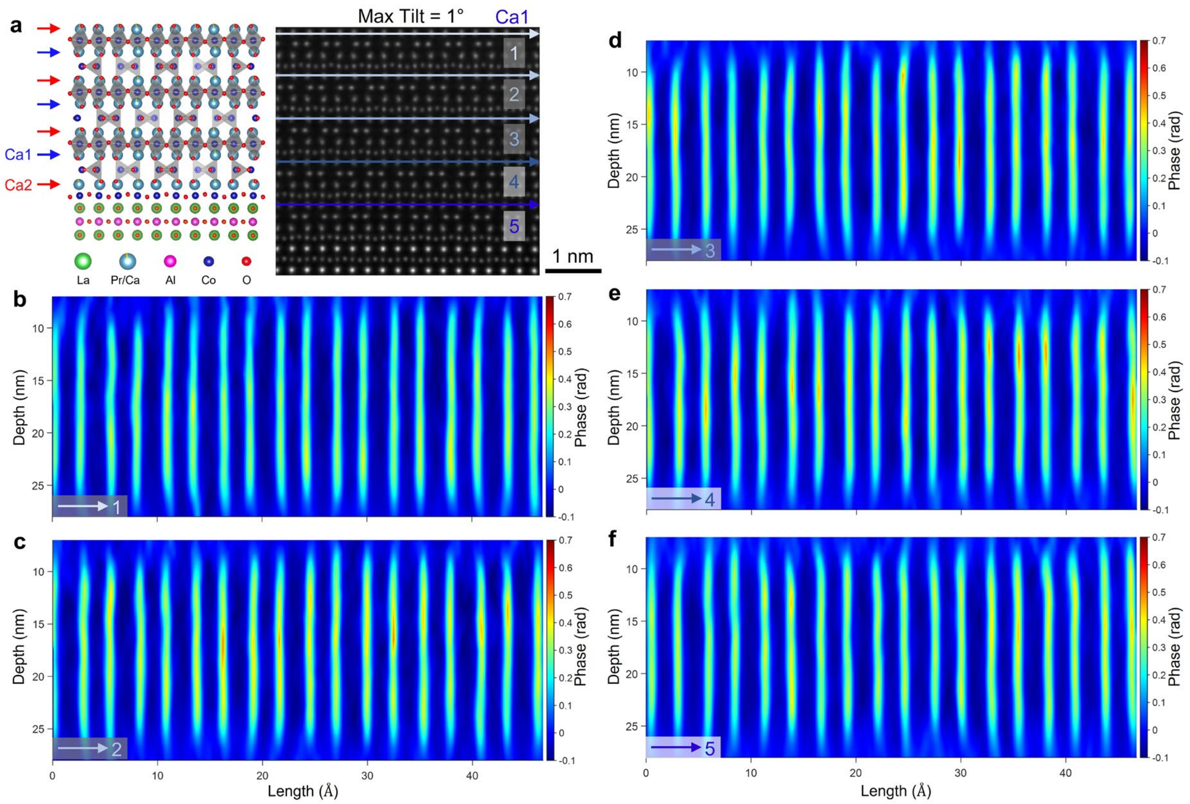

Supplementary Fig. 7| Depth profiles for all Ca1 columns in the TCMEP reconstruction. a, Crystal structure of the $(Pr_{0.05}Ca_{0.95})Co_2O_5$ film (left) and projected phase image reconstructed using TCMEP (right). **b-f**, Depth profiles corresponding to the five arrows along the Ca1 rows marked 1-5 in panel **a**.

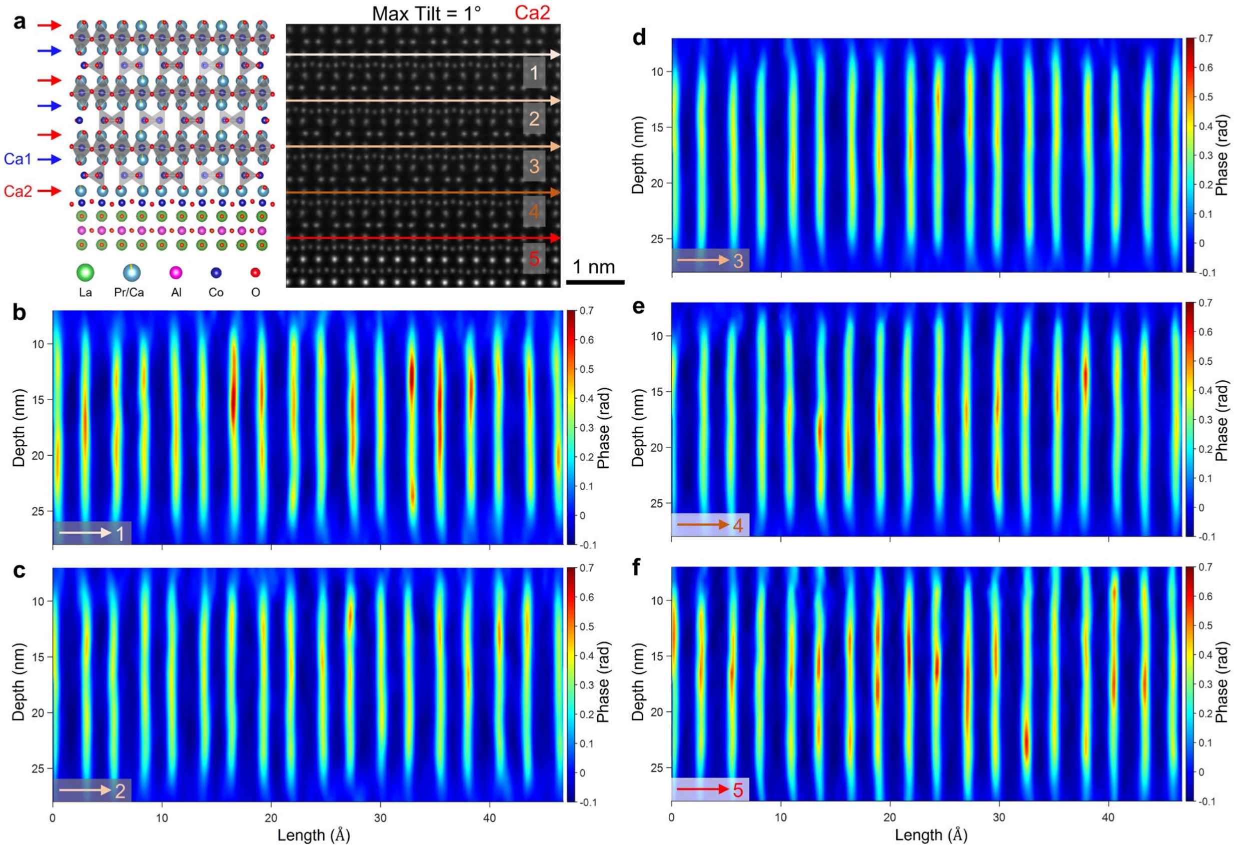

Supplementary Fig. 8| Depth profiles for all Ca2 columns in the TCMEP reconstruction. a, Crystal structure of the $(Pr_{0.05}Ca_{0.95})Co_2O_5$ film (left) and projected phase image reconstructed using TCMEP (right). **b-f**, Depth profiles corresponding to the five arrows along the Ca2 rows marked 1-5 in panel **a**.

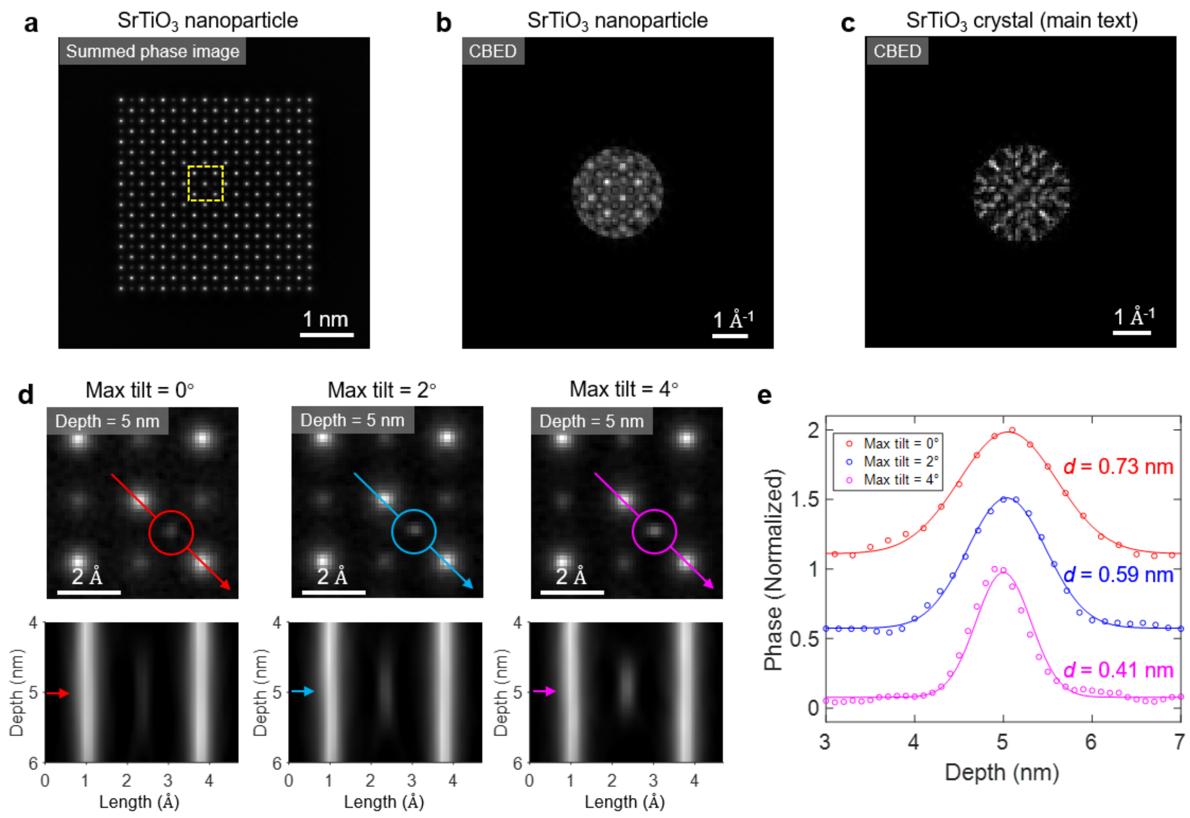

Supplementary Fig. 9| TCMEP results on a simulated weak-scattering $SrTiO_3$ nanoparticle. **a**, Projected phase image of the $SrTiO_3$ nanoparticle (4×4×4 nm^3), reconstructed using MEP. **b-c**, Convergent beam electron diffraction (CBED) patterns for $SrTiO_3$ nanoparticle (**b**) and $SrTiO_3$ crystal in the main text (**c**), corresponding to the weak-scattering regime (**b**) and the strong-scattering regime (**c**) respectively. **d**, Upper panels: reconstructed phase images cropped into the yellow dashed regions in panel **a**, using maximum tilt angles of 0° (left), 2° (middle) and 4° (right). Lower panels: depth profiles along the arrows in the upper panels. The total illumination dose is 2.5×10^6 e/Å^2. **e**, Phase-depth curves for the Sr dopant. The depth resolutions are 0.73 nm, 0.59 nm, and 0.41 nm, respectively.

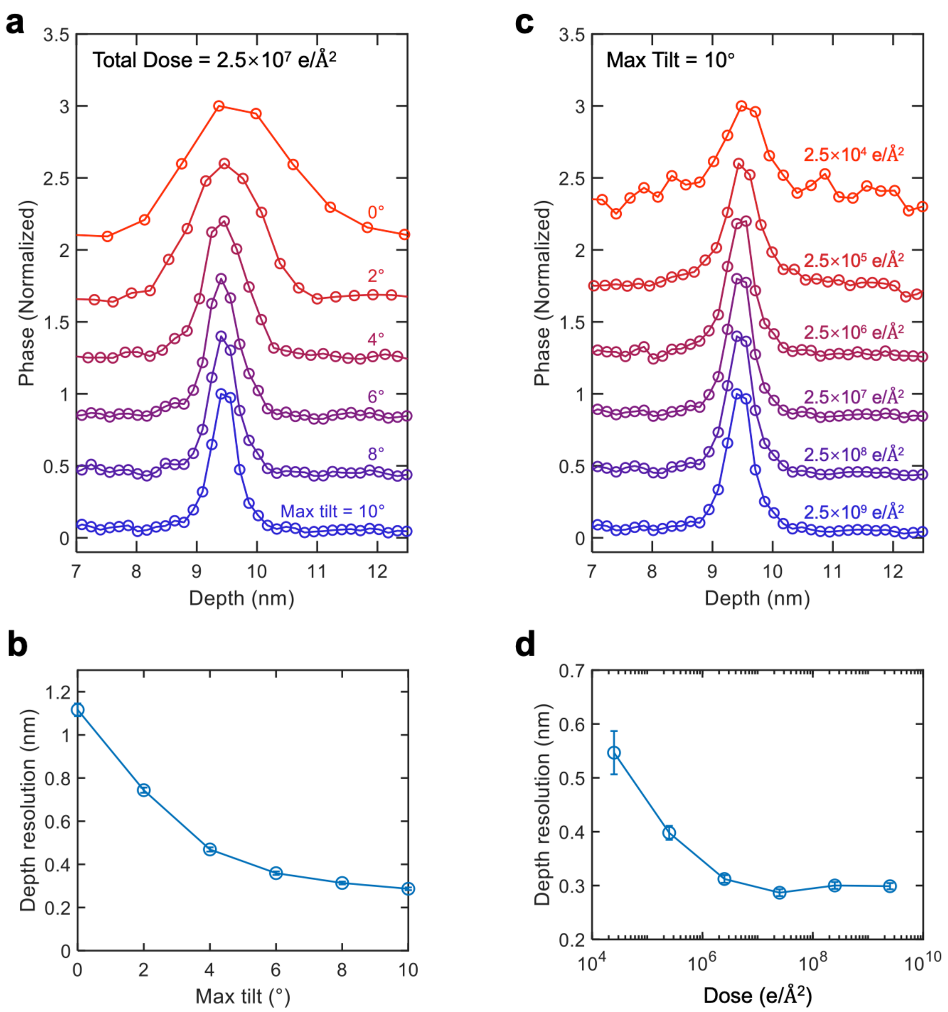

Supplementary Fig. 10| Simulation of TCMEP with larger tilt angles. a, Phase-depth curves of for a Sr dopant under various tilt conditions, while keeping the total illumination dose constant at 2.5×10^7 e/Å^2. Curves are shifted vertically for clarity. **b**, Fitted depth resolution from panel **a** as a function of maximum tilt angle. **c**, Phase-depth curves for the Sr dopant under various total illumination doses, with a fixed maximum tilt of 10°. Curves are shifted vertically for clarity. **d**, Fitted depth resolution from panel **c** as a function of total illumination dose. Error bars are derived from residuals of curve fitting.

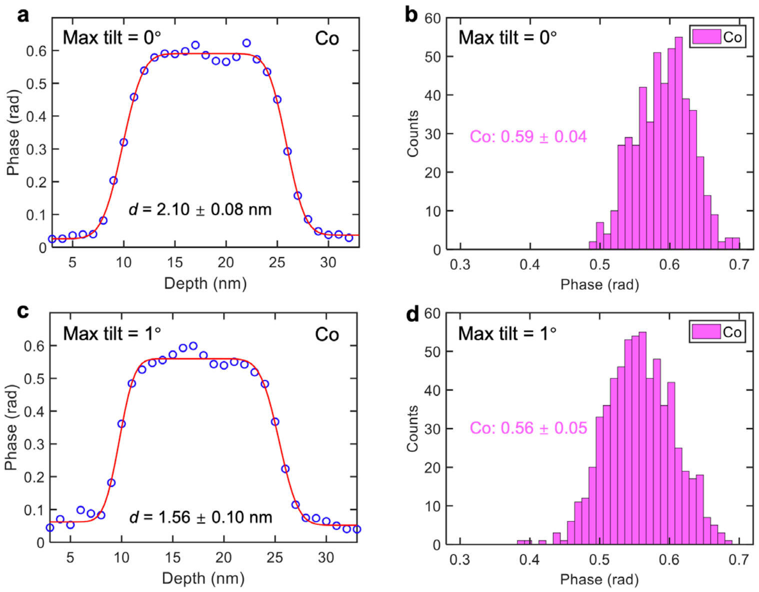

Supplementary Fig. 11| Depth resolution and phase distribution for Co columns in MEP and TCMEP reconstructions of $(Pr_{0.05}Ca_{0.95})_2Co_2O_5$. a, Depth resolution for conventional MEP is ~2.1 nm. **b**, Phase distribution for Co columns shows an uncertainty of 7%. **c**, Depth resolution for TCMEP is ~1.6 nm with a maximum tilt of 1°. **d**, Phase distribution for Co columns shows an uncertainty of 9%, comparable to MEP.

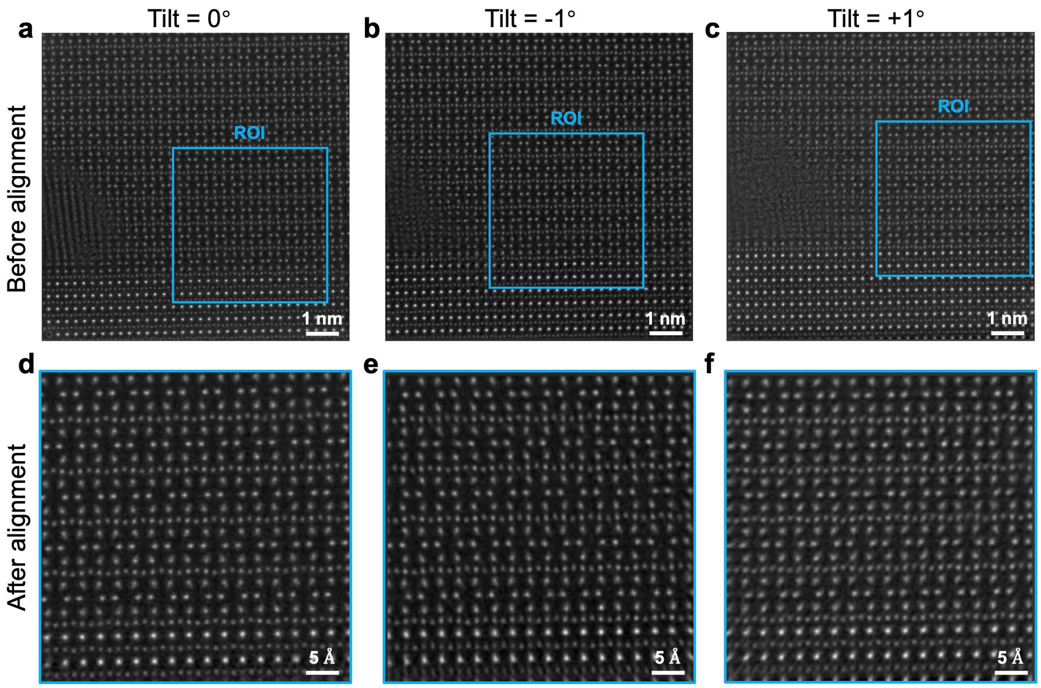

Supplementary Fig. 12| 4D-STEM data alignment prior to TCMEP reconstruction. a-c, MEP reconstruction results for full datasets acquired at tilt angles of 0° (**a**), -1° (**b**) and +1° (**c**). The intentional electron-beam-induced defect regions are used for alignment, with the region of interest (ROI) highlighted by blue squares. **d-f**, MEP reconstruction results for the cropped ROI within the datasets acquired at tilt angles of 0° (**d**), -1° (**e**) and +1° (**f**). The well-aligned datasets are used in the TCMEP reconstruction, while the slight residual misalignments will be further refined during iterations.

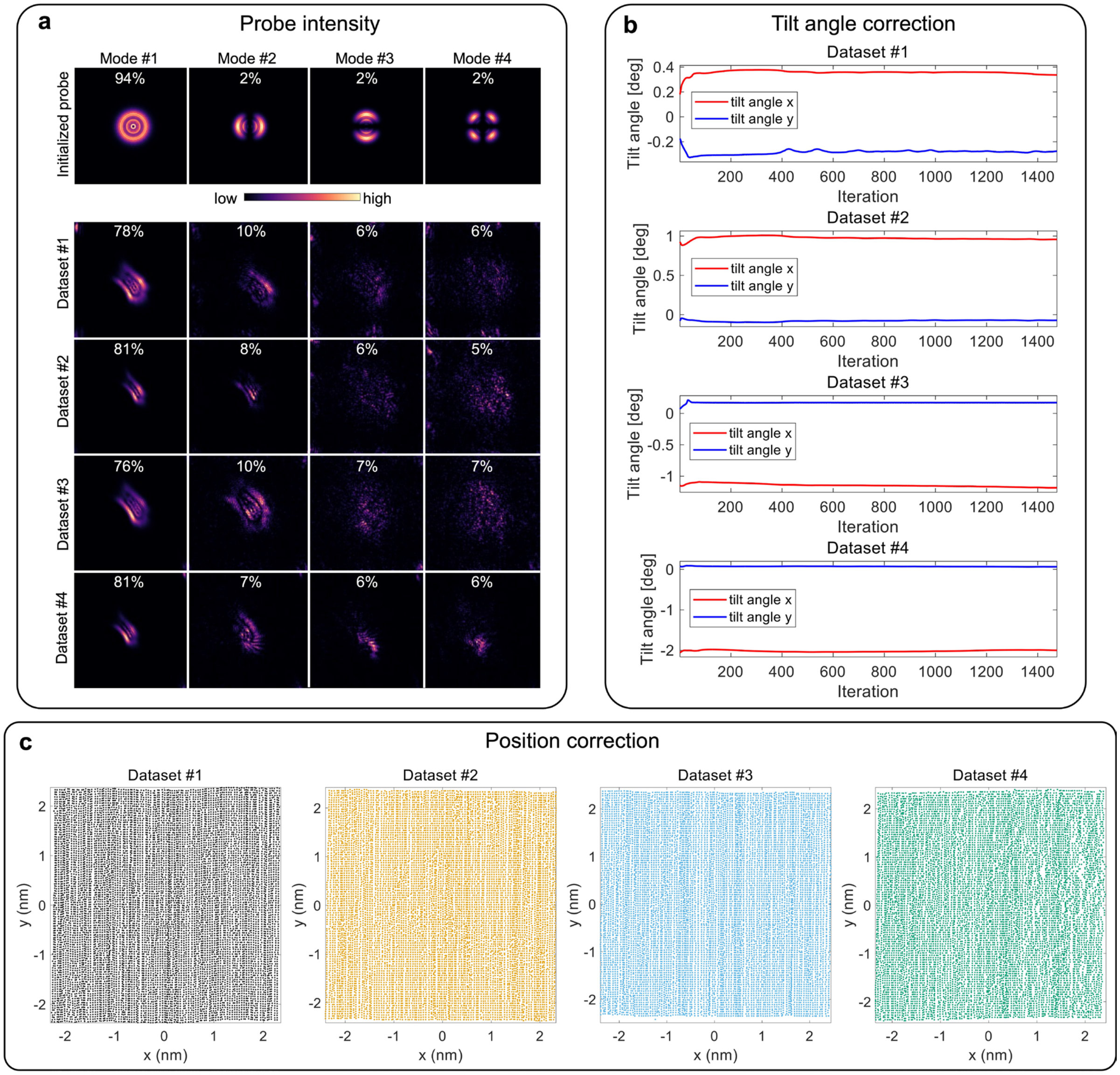

Supplementary Fig. 13| Details of the TCMEP reconstruction for experimental datasets on twisted $SrTiO_3$. a, Initial and reconstructed mixed-state probes for each dataset in TCMEP. **b**, Refinement of tilt angles for each dataset. **c**, Refined probe-positions for each dataset.

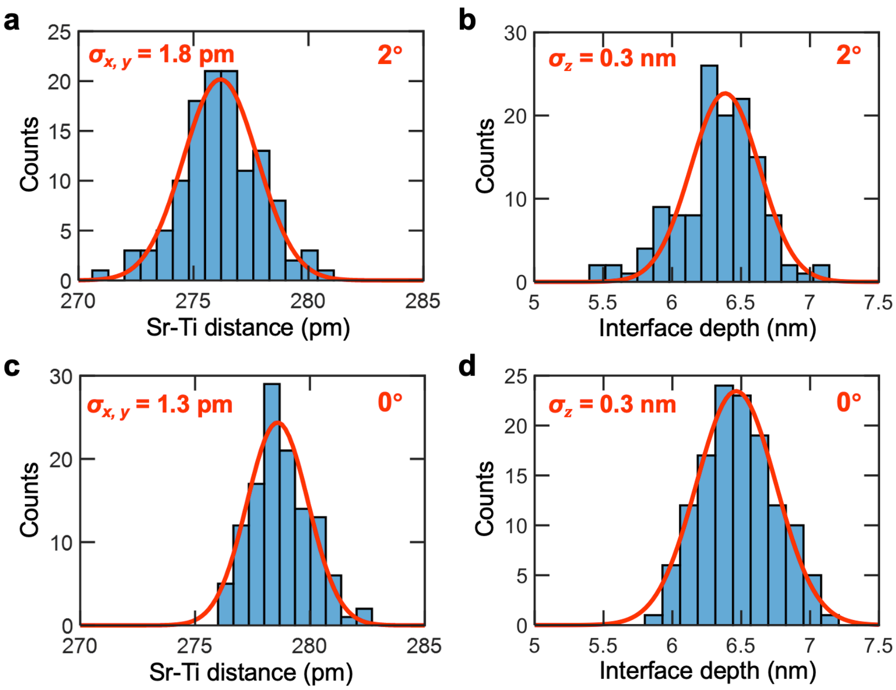

Supplementary Fig. 14| Precision of bond length measurements with TCMEP compared to MEP. a, Histogram of projected Sr-Ti distances from the bottom layer in the TCMEP reconstruction with a maximum tilt of 2°. The average distance is 276.2 ± 1.8 pm, consistent with the $SrTiO_3$ lattice of 276.2 pm. **b**, Histogram of the depth position of the interface in TCMEP reconstruction (maximum tilt 2°), determined by fitting phase-depth curves with error functions. The average depth is 6.3 ± 0.3 nm. **c**, Histogram of projected Sr-Ti distances with MEP. The average distance is 278.8 ± 1.3 pm. **d**, Histogram of the depth position of the interface with MEP. The average depth is 6.5 ± 0.3 nm.

Supplementary Table 1| Reconstruction parameters for data shown in the main text.

	Simulations	Twisted $SrTiO_3$	$(Pr,Ca)_2Co_2O_5$
**Diffraction pixels**	128×128	124×124	124×124
**Scan positions**	26×26	100×100	100×100
**Scan step (Å)**	0.601×0.601	0.458×0.458	0.473×0.473
***k*-space sampling ($Å^{-1}$)**	0.0640×0.0640	0.0330×0.0330	0.0429×0.0429
**Max. collection angle (mrad)**	80.7	40.3	52.4
**No. of iterations**	500	1000	1000
**Batch size**	10×No. of datasets	100 (for θ_{max}=0°)<br>200 (for θ_{max}=1°)<br>400 (for θ_{max}=2°)	100 (for θ_{max}=0°)<br>200 (for θ_{max}=1°)
**beta_LSQ**	1.0	2.0	2.0
**Noise model**	Amplitude likelihood		
**sigma_PSF (pixel)**	0.0	1.0	1.0
**No. of probe modes**	2	4	4
**Object sampling (Å)**	0.122×0.122×[4.0 to 1.3]	0.236×0.236×4.0	0.182×0.182×10.0
**Object size (pixel)**	454×454×[40 to 120]	372×372×40	455×455×30
**Layer regularization**	0.10	0.30 (for θ_{max}=0°)<br>0.15 (for θ_{max}=1°, 2°)	0.30 (for θ_{max}=0°)<br>0.10 (for θ_{max}=1°)
