# Supplementary material for: Sub-nanometer depth resolution and single dopant visualization achieved by tilt-coupled multislice electron ptychography
Source: Nat Commun. 2025 Jan 31;16:1219. doi: 10.1038/s41467-025-56499-1 (PMC11785980; doi:10.1038/s41467-025-56499-1)
Supplement: Supplementary file 2 — Description of Additional Supplementary Files [file 41467_2025_56499_MOESM2_ESM.pdf]

Description of Additional Supplementary Files

Supplementary Movie 1| Depth dependence of atomic displacements and Pr dopants overlaid on the phase images of each slice.

Upper panel: TCMEP-reconstructed slice images of the $(Pr_{0.05}Ca_{0.95})Co_2O_5$ thin film, spanning depths from 13 nm to 21 nm relative to the sample top surface. Lower panel: Corresponding peak phase of Ca columns for each slice image. Cyan arrows indicate atomic displacements overlaid on the projected phase image, with a reference displacement magnitude of 20 pm provided for comparison. This video reveals the clear relationship between Pr dopants (indicated by bright yellow squares) and atomic displacements. Note that slice images at depths below 13 nm or above 21 nm are dominated by amorphous or vacuum layers, preventing the extraction of atomic positions.
